# A Log-Ratio Biplot Approach for Exploring Genetic Relatedness Based on Identity by State

**DOI:** 10.3389/fgene.2019.00341

**Published:** 2019-04-24

**Authors:** Jan Graffelman, Iván Galván Femenía, Rafael de Cid, Carles Barceló Vidal

**Affiliations:** ^1^Department of Statistics and Operations Research, Technical University of Catalonia, Barcelona, Spain; ^2^Department of Biostatistics, University of Washington, Seattle, WA, United States; ^3^Department of Computer Science, Applied Mathematics and Statistics, University of Girona, Girona, Spain; ^4^Genomes For Life - GCAT Lab, Institute for Health Science Research Germans Trias i Pujol (IGTP), Badalona, Spain

**Keywords:** allele sharing, composition, identity by state, identity by descent, log-ratio transformation

## Abstract

The detection of cryptic relatedness in large population-based cohorts is of great importance in genome research. The usual approach for detecting closely related individuals is to plot allele sharing statistics, based on identity-by-state or identity-by-descent, in a two-dimensional scatterplot. This approach ignores that allele sharing data across individuals has in reality a higher dimensionality, and neither regards the compositional nature of the underlying counts of shared genotypes. In this paper we develop biplot methodology based on log-ratio principal component analysis that overcomes these restrictions. This leads to entirely new graphics that are essentially useful for exploring relatedness in genetic databases from homogeneous populations. The proposed method can be applied in an iterative manner, acting as a looking glass for more remote relationships that are harder to classify. Datasets from the 1,000 Genomes Project and the Genomes For Life-GCAT Project are used to illustrate the proposed method. The discriminatory power of the log-ratio biplot approach is compared with the classical plots in a simulation study. In a non-inbred homogeneous population the classification rate of the log-ratio principal component approach outperforms the classical graphics across the whole allele frequency spectrum, using only identity by state. In these circumstances, simulations show that with 35,000 independent bi-allelic variants, log-ratio principal component analysis, combined with discriminant analysis, can correctly classify relationships up to and including the fourth degree.

## 1. Introduction

**Figure 1:**
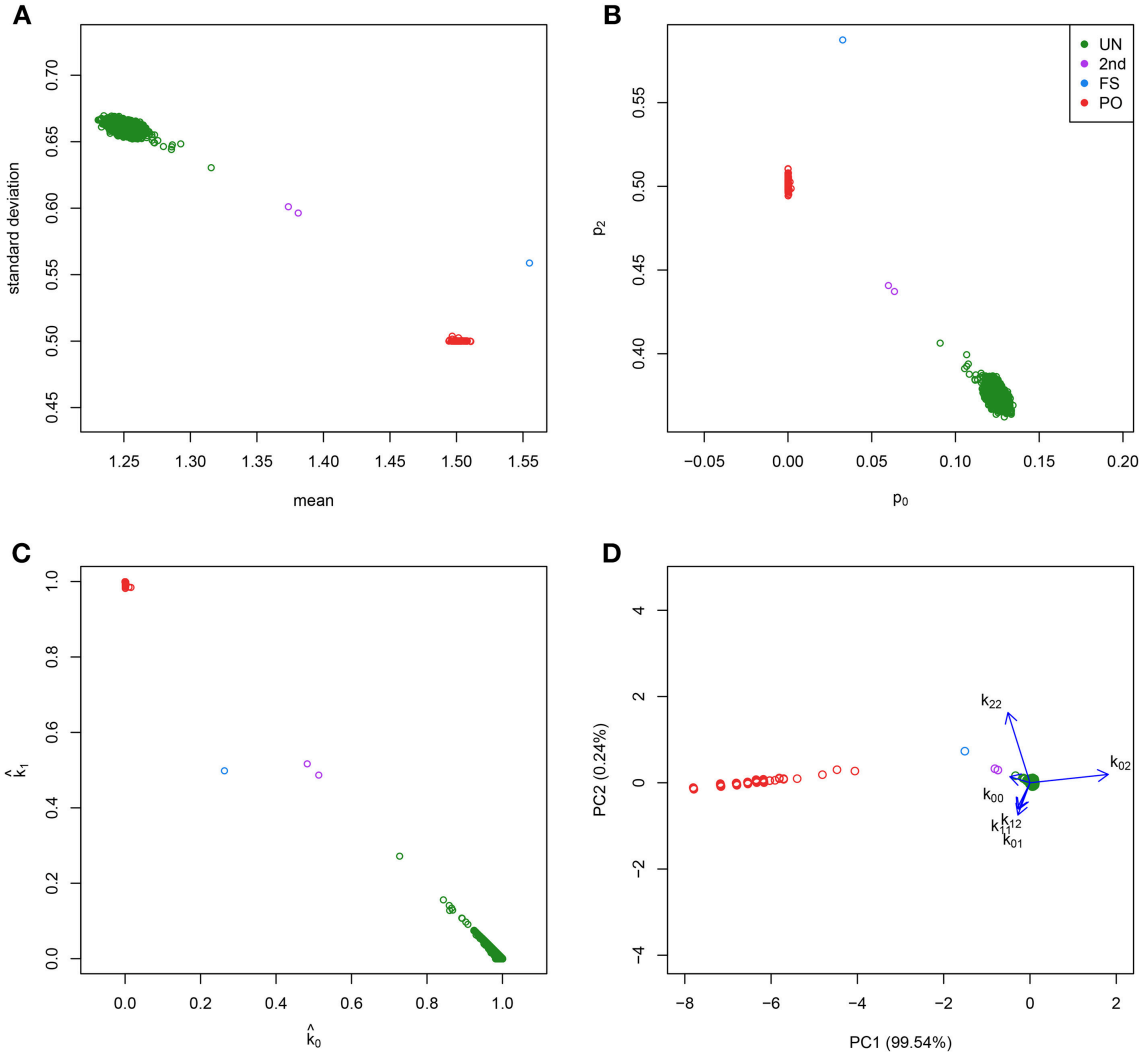
Classical graphics for relatedness research and log-ratio PCA biplot. Plots show the CEU sample of the 1000G project. IBS/IBD statistics were calculated over a set of 26,081 complete, LD-pruned autosomal SNPs with MAF above 0.4, and HWE exact test *p*-value above 0.05. **(A)** Scatterplot of the mean and standard deviation of the number of IBS alleles. **(B)** Scatterplot of the fraction of variants sharing two (*p*_2_) against the fraction sharing zero (*p*_0_) IBS alleles. **(C)** Scatterplot of the estimated probability of sharing one (${\hat{k}}_{1}$) against the estimated probability of sharing zero (${\hat{k}}_{0}$) IBD alleles. **(D)** log-ratio PCA biplot.

The detection of pairs of related individuals in genomic databases is important in many areas of genetic research. In population-based gene-disease association studies, the assumption of independent observations which is usually made in the statistical modeling of the data, may be violated due to related individuals. Cryptic relatedness can lead to an increased false positive rate in association studies, in particular if related individuals are oversampled (Voight and Pritchard, 2005). In conservation genetics, unrelated individuals are carefully selected in breeding programs in order to maximize genetic diversity (Oliehoek et al., 2006). In quality control of genetic variants produced by high-throughput techniques, accidental duplication of samples in genetic studies can be detected by a relatedness analysis (Abecasis et al., 2001). In ecology, samples of species often contain an excess of close relatives. This can lead to biased estimates of population-genetic parameters, lower the precision of their estimates, and inflated type 1 error rates of tests for genetic equilibria (Wang, 2018). In practice, most relatedness investigations are based on allele-sharing statistics such as the average number of identical-by-state (IBS) alleles shared by a pair of individuals over a set of loci, or by estimating the probabilities of sharing 0, 1, or 2 alleles identical-by-descent (IBD; Thompson, 1975, 1991), known as Cotterman's coefficients (Cotterman, 1941). Plots of these sharing statistics typically show clusters that correspond to unrelated pairs (UN), parent-offspring pairs (PO), full sibs (FS), half sibs (HS), monozygotic twins (MZ), avuncular pairs (AV), first cousins (FC), grandparent-grandchild (GG), or more remote relationships (see Figures 1A–C).

All these methods collapse the data to two statistics, that can summarize relatedness in two dimensions. Classical plots are the mean vs. the standard deviation of the shared number of alleles over loci [the (*m, s*) plot, see Figure 1A], the fractions of loci for which a pair of individuals shares 0 or 2 IBS alleles [the (*p*_0_, *p*_2_) plot, see Figure 1B], or the estimated probabilities of sharing 0 or 1 allele IBD [the ($\hat{k}0$, $\hat{k}1$) plot, see Figure 1C]. However, all allele sharing statistics are estimated from the genotype data. For a pair of individuals with bi-allelic variants, there exist six possible pairs of genotypes, and their counts over the *k* variants determine the IBS allele sharing statistics. From this perspective, the observed genotype sharing data consists of vectors of six elements, that, when expressed in percentage form, occupy a five dimensional space. This suggests that the classical approaches of collapsing the data into two dimensions by plotting the summary statistics may not extract all information about relatedness that is present in the data. In this paper we propose to explore the data in five dimensions by using log-ratio principal component analysis (PCA), which is specially designed for analyzing compositional data (Aitchison, 1983). A log-ratio PCA allows us to construct comprehensive biplots that uncover the main relatedness features of the data.

Biplots are widely used in genetic research, in particular for the graphical representation of quantitative traits of genotypes in plant genetics (Anandan et al., 2016; Pandit et al., 2017; Sharma et al., 2018). In relatedness research, a PCA of bi-allelic genetic variants, coded in 0, 1, 2 format (for AA, AB, and BB respectively) is often used to investigate the existence of population substructure, that is, remote genetic relatedness. The plots obtained by this kind of PCA are, in principle, biplots, though often the genetic variants are omitted in such plots because there are too many of them. Substructure is also often investigated by multidimensional scaling (MDS) of allele sharing distances between individuals. The resulting MDS maps only represent individuals, and some authors prefer the term monoplots for such graphics (Gower et al., 2011). If MDS is based on the Euclidean distances, then a covariance-based PCA and MDS are in fact equivalent (Mardia et al., 1979). The MDS plots, PCA biplots without variable vectors for the genetic variants, are particularly popular in substructure investigations in human genetics (Jakobsson et al., 2008; Sabatti et al., 2009; Pemberton et al., 2010, 2013; Wang et al., 2010).

The biplot approach proposed in this paper differs from the classical applications described above in several ways. We propose a biplot of the genetic data of *pairs* of individuals, that represents artificially related pairs of a reference set of given familial relationships, generated by a respampling of the genetic data. The empirically observed pairs are used in a supplementary way, and are projected onto the reference biplot. The data matrix used in this biplot is not a (0, 1, 2) genetic data matrix, neither a distance matrix of allele sharing distances, but consists of vectors of counts of genotype patterns [(AA,AA), (AA,AB), etc.] which we treat as compositions, and we therefore use a log-ratio approach. More details are given in the section 2 below.

An important additional advantage of using log-ratio PCA in this context is that it allows us to explore the data iteratively with a *peel and zoom* procedure. A first log-ratio PCA may clearly reveal a cluster of FS pairs. Once identified, the corresponding pairs can be removed from the database, and log-ratio PCA can be repeated on the remaining pairs. The second analysis will focus more closely on more remote relationships that may be present in the database, and thereby act as a magnifying glass for the latter. The aforementioned classical graphics do not have this property, as they are invariant under removal of a relationship category.

The remainder of this paper is organized as follows. In the section 2 we provide background on relatedness research and log-ratio PCA, and show how to construct biplots that are useful for relatedness research. In the section 3 we study the discriminative power of log-ratio PCA and compare this with the classical plots in a simulation study. We also describe two empirical examples of our method with data from two different population-based datasets; a next generation sequencing dataset from the 1,000 Genomes Project (The 1000 Genomes Project Consortium, 2015) and a genome-wide SNP array technology dataset from the GCAT Genomes For Life Cohort Study of the Genomes of Catalonia (Galván-Femenía et al., 2018; Obón-Santacana et al., 2018). A discussion finishes the paper.

## 2. Methods

We first summarize some basic methods for relatedness research (section 2.1), then give a brief account of log-ratio PCA (section 2.2), and finally show how log-ratio PCA can be used in relatedness research (section 2.2).

### 2.1. Relatedness Research

We briefly review some fairly standard procedures that are currently used in relatedness research. Relatedness investigations are focused on the extent to which alleles are shared between individuals. Two individuals can share 0, 1, or 2 alleles for any autosomal variant. Alleles can be identical by state (IBS) or identical by descent (IBD). A pair of individuals share IBS alleles if they match irrespective of their provenance; whereas they share IBD alleles only if they come from a common ancestor. Table 1 shows all the possible combinations of the IBS alleles shared for a pair of individuals at a biallelic variant. Considering *k* biallelic variants, each pair of individuals has a vector of 0, 1, and 2 IBS counts of length *k*. In IBS studies, the means (*m*) and standard deviations (*s*) of the vector of the IBS allele counts (Abecasis et al., 2001), or the proportions of variants sharing 0, 1, and 2 IBS alleles (denoted *p*_0_, *p*_1_, and *p*_2_ respectively, Rosenberg, 2006) can be plotted (see Figures 1A,B). These plots reveal characteristic clusters corresponding to MZ, PO, FS, UN, and other pairs. Alternatively, in an IBD based approach, the probability of sharing 0, 1, or 2 IBD alleles for a pair of individuals (usually denoted by *k*_0_, *k*_1_, and *k*_2_ and referred to as Cotterman's coefficients) can be represented in a scatterplot (see Figure 1C, Nembot-Simo et al., 2013). The Cotterman coefficients can be estimated by the method of moments (Purcell et al., 2007), maximum likelihood (Thompson, 1991; Milligan, 2003; Weir et al., 2006), or robust estimation methods (the KING program, Manichaikul et al., 2010). In IBD studies, reference values for the standard relationships are available (see Table 2). Related pairs can also be distinguished, albeit at lower resolution, by using the co-ancestry coefficient defined as θ = *k*_1_/2+*k*_2_ or the kinship coefficient defined as ϕ = θ/2. Galván-Femenía et al. (2017) give an overview of graphics used in relatedness research. Figure 1 shows a panel plot of some standard graphics used in IBS and IBD studies for all the pairs of individuals from the CEU population of the 1.000 Genomes project. These plots distinguish UN, PO, FS, and second degree pairs. Alternatively, a Markov-chain approach with the calculation of likelihood ratios for putative and alternative relationship has been developed by Epstein et al. (2000; the Relpair program) and by McPeek and Sun (2000; the Prest-plus program). Throughout this paper we employ the classical notion of degree of relationship, shown in the second column of Table 2, with PO and FS being considered first degree, HS, GG and AV, second degree, FC third degree, first cousins once removed fourth degree, second cousins fifth degree and second cousins once removed sixth degree, and so on.

**Table 1:** Number of IBS alleles for possible combinations of genotypes.

	**AA**	**AB**	**BB**
AA	2	1	0
AB	1	2	1
BB	0	1	2

**Table 2:** IBD probabilities for standard relationships.

			**IBD probabilities**		

**Type of relative**	**R**	**ϕ**	***k*_0_**	***k*_1_**	***k*_2_**
Monozygotic twins (MZ)	0	1/2	0	0	1
Full-siblings (FS)	1	1/4	1/4	1/2	1/4
Parent-offspring (PO)	1	1/4	0	1	0
Half-siblings ∣ grandchild-grandparent ∣	2	1/8	1/2	1/2	0
niece/nephew-uncle/aunt (HS,GG,AV)					
First cousins (FC)	3	1/16	3/4	1/4	0
Unrelated (UN)	∞	0	1	0	0

*Degree of relationship (R), kinship coefficient (ϕ), and probability of sharing zero, one, or two alleles identical by descent (k_0_, k_1_, k_2_)*.

### 2.2. Log-Ratio Principal Component Analysis

Aitchison (1983) proposed log-ratio principal component analysis (PCA) for the exploration of compositional data. Many successful applications of log-ratio PCA have been described in the literature, notably in geology. We briefly summarize log-ratio PCA and biplot construction (see Pawlowsky-Glahn et al., 2015 for a comprehensive account). Log-ratio PCA is usually performed by applying the centered log-ratio transformation to the compositional data, and we will follow that approach here. Let **X** be a matrix with *n* compositions in its rows, and having *D* parts (columns). Compositional data can be defined as strictly positive vectors for which the information of interest is in the ratios between the components (Aitchison, 1986). We consider the centered log-ratio transformation (clr) of a composition **x** (a row of **X**) given by

(1) $$\text{clr}(\text{x})=\left[\ln\left(\frac{x_{1}}{\text{gm}(\text{x})}\right),\text{ln}\left(\frac{x_{2}}{\text{gm}(\text{x})}\right),\cdots ,\text{ln}\left(\frac{x_{D}}{\text{gm}(\text{x})}\right)\right],$$

where gm(**x**) is the geometric mean of the components of the composition **x**. Let **X**_ℓ_ be the log transformed compositions, that is **X**_ℓ_ = ln (**X**) with the natural logarithmic transformation applied element-wise. The clr transformed data can be obtained by just centering the rows of this matrix, using the centering matrix ${\text{H}}_{r}=\text{I}–\frac{1}{D}11^{\prime }$. Then

(2) $$\begin{array}{r} {\text{X}}_{\text{clr}}={\text{X}}_{\ell }{\text{H}}_{r}, \end{array}$$

The rows of **X**_clr_ are subject to a zero sum constraint because **H**_*r*_**1** = **0**. If there are no additional linear constraints, then **X**_clr_ will have rank *D*−1. We now column-center the clr transformed data, producing a double-centered data matrix that has zero column and row means:

(3) $$X_{c\text{clr}}=H_{c}X_{\text{clr}}=H_{c}X_{\ell }H_{r},$$

where **H**_*c*_ is the centering matrix ${\text{H}}_{c}=\text{I}-\left(1/n\right)11^{\prime }$. Matrix **X**_*c*clr_ is used as the input for a classical principal component analysis. We perform PCA by the singular value decomposition:

(4) $$\begin{array}{r} {\text{X}}_{c\text{clr}}=\text{U}\text{D}{\text{V}}^{\prime }={\text{F}}_{p}{{\text{G}}_{s}}^{\prime }, \end{array}$$

with **F**_*p*_ = **UD** and **G**_*s*_ = **V**. Matrix **F**_*p*_ contains the principal components, and its first two columns contain the biplot coordinates of the compositions. The columns of **G**_*s*_ are the eigenvectors of the covariance matrix of **X**_*c*clr_, its first two columns contain the biplot coordinates of the parts of the compositions. We use sub-indexes *p* and *s* to distinguish principal and standard biplot coordinates. We will need to project supplementary compositions onto a given biplot (see section 3). This can be accomplished by regression (Graffelman and Aluja-Banet, 2003). The biplot coordinates, ${\tilde{\text{F}}}_{p}$, of a matrix of supplementary compositions, **Y**, can be found as

(5) $$\begin{array}{r} {\tilde{\text{F}}}_{p}={\left({{\text{G}}_{s}}^{\prime }{\text{G}}_{s}\right)}^{-1}{{\text{G}}_{s}}^{\prime }{\text{Y}}_{c\text{clr}}, \end{array}$$

where **Y**_*c*clr_ contains the clr-transformed supplementary compositions, but centered with respect to the compositions in **X**, that is

(6) $$\begin{array}{r} {\text{Y}}_{c\text{clr}}={\text{Y}}_{\text{clr}}-\frac{1}{n}11^{\prime }{\text{X}}_{\text{clr}}. \end{array}$$

We will construct a biplot of genotypic reference compositions by using Equation (4), and project empirical genotype compositions onto the biplot by using Equations (5) and (6).

### 2.3. Log-Ratio PCA of Genotype Sharing Data

For bi-allelic variants with alleles A and B, there exist six possible pairs of genotypes whose counts over *k* variants can be laid out in a triangular array shown in Table 3, where *k*_*ij*_ refers to the number of variants that have *i* B alleles for one individual, and *j* B alleles for the other individual. Consequently, each pair can be represented by a vector of six counts which can be expressed as a composition by division by its total (closure):

(7) $$\begin{array}{r} \text{x}=\left(k_{00},k_{10},k_{20},k_{11},k_{21},k_{22}\right)/k. \end{array}$$

The total number of variants is given by $k=\underset{i\geq j}{\sum }k_{ij}$. For PO pairs this vector has, in theory, a structural zero, *k*_20_ = 0, because PO pairs share at least one IBS allele. However, for empirical data *k*_20_ = 0 is, with large *k*, almost never observed due to the existence of some mutations and genotyping error. Given *n* individuals, we construct matrix **X** with $q=\frac{1}{2}n\left(n-1\right)$ pairs in its rows, and propose to study relatedness by a log-ratio PCA of this *q*×6 matrix of compositions. This will allow the construction of a biplot, where each pair of individuals is represented by a point, and each part of the clr transformed composition by a vector. A drawback of the representation of pairs of individuals in a log-ratio PCA biplot is that the type of relationship cannot be inferred if it is undocumented. Without additional analysis one does not know for sure whether observed clusters correspond to FS, HS, or other pairs. We resolve this by first identifying a subset of approximately unrelated individuals in the database, having a co-ancestry coefficient with other individuals that is below 0.05. We next simulate pairs of related individuals of known relationships by constructing pedigrees from this subset, applying the Mendelian inheritance rules. For example, PO pairs are simulated by first drawing two parents at random from the unrelated subset. A child is then simulated by drawing one allele at random from both these parents. The process is repeated in order to generate many random PO pairs. FS, HS, and pairs of other relationships are simulated in an analogous manner. This process is based on a re-sampling the alleles of the observed individuals. The artificially generated data set forms a *reference set* or *training set* against which the empirically observed data can be compared. This reference set is generated conditionally on the allele frequencies of the observed sample. We now first apply log-ratio PCA to the pairs of the reference set (**X**), and construct a biplot of the reference set. The empirically observed pairs (**Y**) are projected onto this PCA biplot and their relationship is inferred, according to which simulated type of relationship is most close to the empirical pair. This can be done in a quantitative way by classifying all empirical pairs with linear discriminant analysis (LDA) (Johnson and Wichern, 2002), using the simulated pairs as a training set.

**Table 3:**
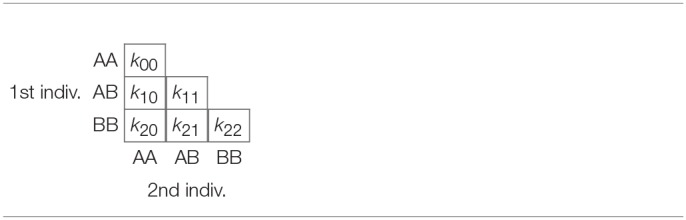
Lower triangular matrix layout with counts for all possible genotype pairs.

*All possible genotype pairs for a bi-allelic genetic variant. k_ij_ represents the number of genetic variants with i and j B alleles for a pair of individuals*.

## 3. Results

In this section we first validate the proposed methodology with some simulations, comparing the log-ratio PCA approach with the well-known aforementioned (*m, s*), (*p*_0_, *p*_2_), and ($\hat{k}0$, $\hat{k}1$) plots, and then show two examples with empirical genetic data.

### 3.1. Simulations

**Figure 2:**
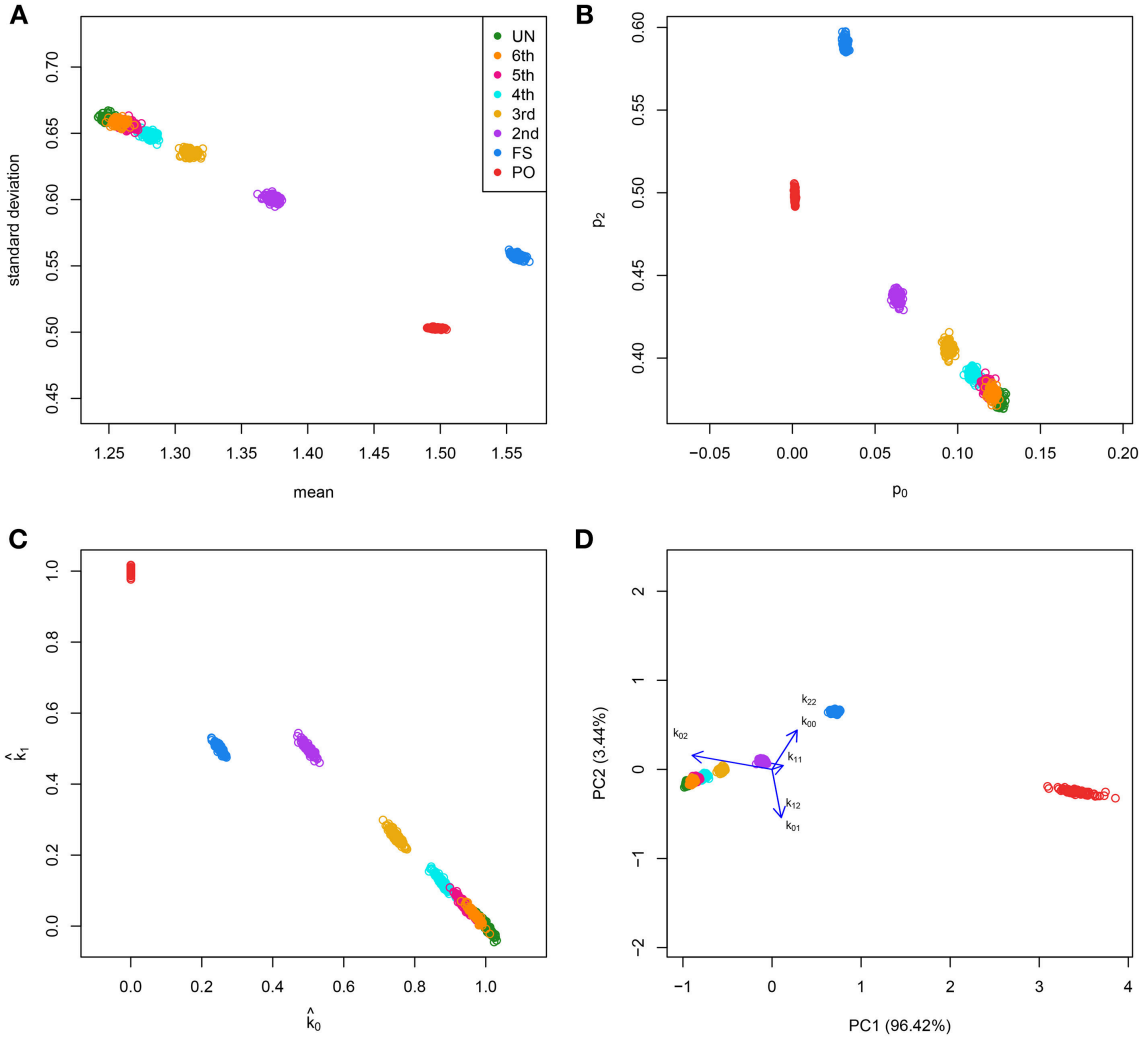
Classical graphics and log-ratio PCA biplot for simulated samples. 100 pairs of each type of relationship [UN, sixth, fifth, fourth, third (FC), second (HS), FS, and PO] were generated using 35,000 independent bi-allelic variants with minor allele frequencies of 0.5, assuming Hardy-Weinberg equilibrium. (A) Scatterplot of the mean and standard deviation of the number of IBS alleles. (B) Scatterplot of the fraction of variants sharing two (*p*_2_) against the fraction sharing zero (*p*_0_) IBS alleles. (C) Scatterplot of the estimated probability of sharing one (${\hat{k}}_{1}$) against the estimated probability of sharing zero (${\hat{k}}_{0}$) IBD alleles. (D) log-ratio PCA biplot.

**Figure 3:**
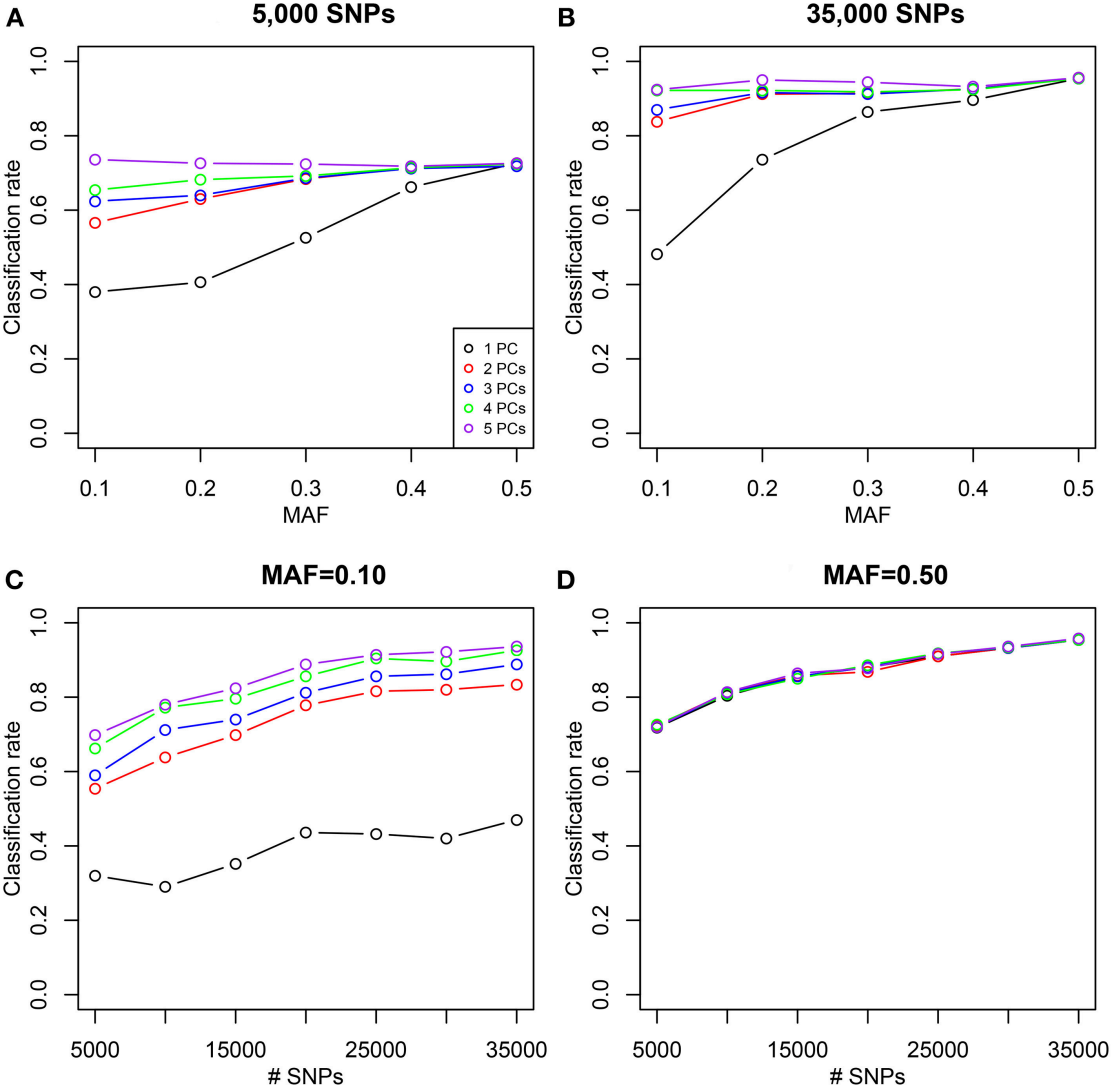
Classification rate of log-ratio PCA combined with LDA for simulated samples. Classification rate for a varying number of principal components (PCs). Classification rates were obtained using 100 pairs of each type of relationships (UN, sixth, fifth, fourth, and third) using independent variants simulated assuming Hardy-Weinberg equilibrium. (A,B) Classification rates are shown as a function of the MAF for 5,000 and 35,000 SNPs. (C,D) Classification rates are shown as a function of the number of SNPs of a given MAF (0.10 and 0.50).

We simulated 35,000 independent genetic bi-allelic variants by sampling from a multinomial distribution under the Hardy-Weinberg assumption, using a minor allele frequency (MAF) of 0.5 for all variants. Using Mendelian inheritance rules, 100 independent pairs of each type of relationship were simulated. We assume a homogeneous population without mutation and genotyping error, generating simulated data sets that are free of Mendelian inconsistencies. The classical plots and the log-ratio PCA biplot of a simulation are shown in Figure 2. This figure shows that first and second degree pairs are easily identified by all methods. We will therefore focus on third and higher degree relationships which are harder to distinguish as they tend to blur in the plots. We investigated the effect of MAF and number of SNPs on the classification rate of our procedure, using different numbers of principal components for classification of third through sixth degree pairs (100 of each). Figure 3 shows the classification rates obtained as a function of the minor allele frequency (MAF), the number of SNPs and the number of principal components used. These figures show, as expected, that the classification rate increases with the MAF and the number of SNPs. The simulations show that all five components are needed at low MAF, where more components increase the classification rate. At high MAF (0.40–0.50) there is little or no benefit in using more than two components. With 35,000 SNPs at 0.50 MAF the classification rate is around 95% irrespective of the number of components. With 35,000 SNPs at 0.10 MAF the classification rate varies from below 50% with one component through 93% using all five components. We report the false positive rates in Table S1; No UN or 6th degree individuals were misclassified as 4th degree or lower, and only 1.8% of the 5th degree pairs are misclassified as 4th degree. The simulations show that IBS based log-ratio PCA can discriminate higher degree relationships if a sufficient number of independent highly polymorphic variants is available. In the light of the simulations, we decided to use three principal components for classification with high MAF variants for the empirical data sets described in section 3.3.

### 3.2. Method Comparison

**Figure 4:**
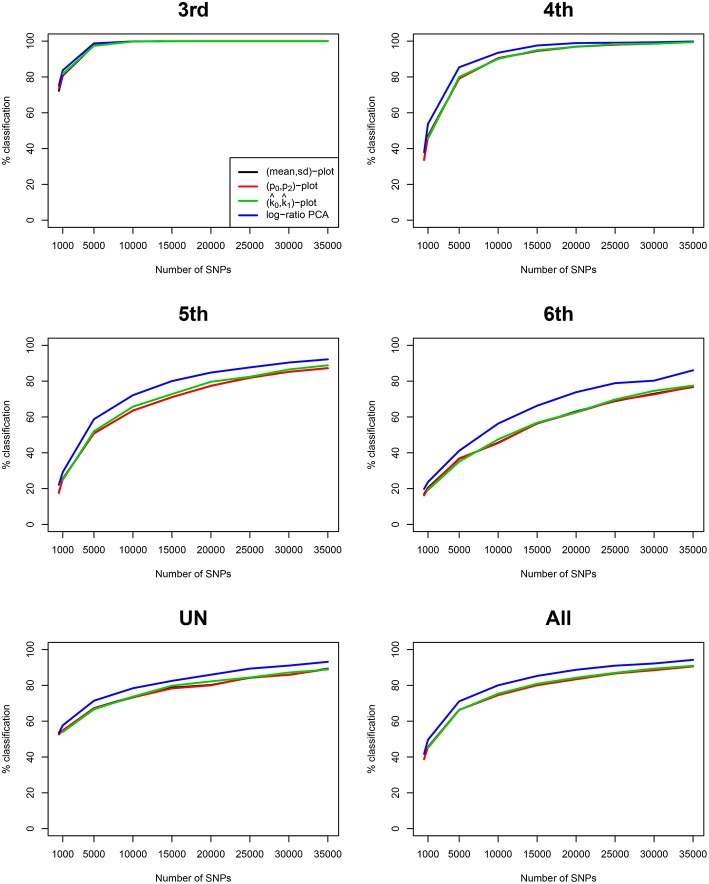
Classification rates for different methods vs. number of SNPs. Classification rates for the different degrees of relationship (third, fourth, fifth, sixth, UN, and All) are shown for four methods, using five principal components. Classification rate profiles for the (*m, s*) plot and the (*p*_0_, *p*_2_) plot virtually coincide. The last panel All refers to the classification rate for third through UN relationships jointly. Rates are shown as a function of the number of SNPs with MAF 0.50, and were obtained by linear discriminant analysis. 100 pairs of each type of relationship (UN, fifth, fourth, third, second, FS, and PO) were generated assuming Hardy-Weinberg equilibrium.

**Figure 5:**
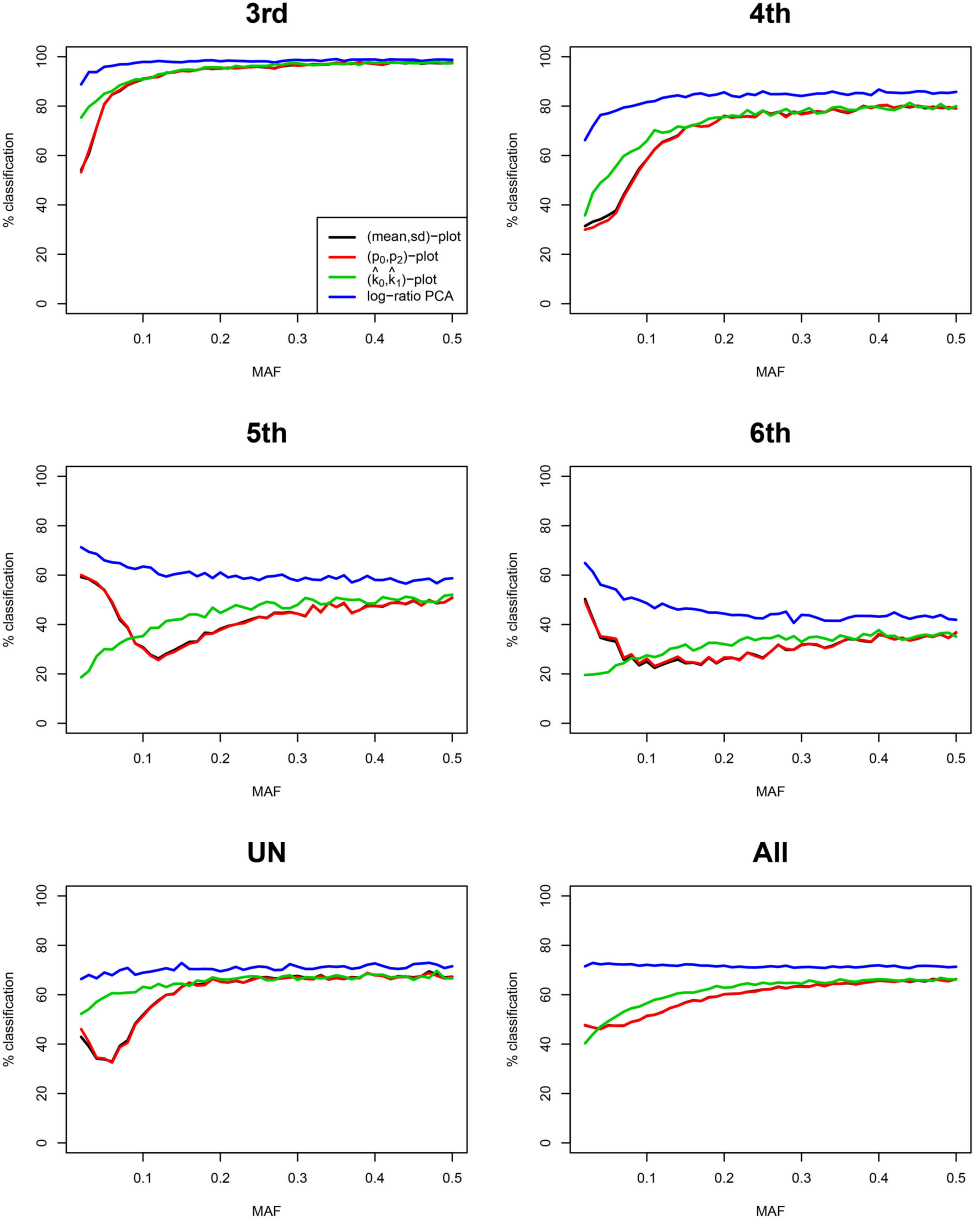
Classification rates for different methods vs. MAF. Classification rates for the different degrees of relationship (third, fourth, fifth, sixth, UN, and All) are shown for four methods, using three principal components. Classification rate profiles for the (*m, s*) plot and the (*p*_0_, *p*_2_) plot virtually coincide. The last panel All refers to the classification rate for third through UN relationships jointly. Rates are shown, using 5,000 SNPs, as a function of the MAF, and were obtained by linear discriminant analysis. 100 pairs of each type of relationship (UN, sixth, fifth, fourth, and third) were generated assuming Hardy-Weinberg equilibrium.

We compare our method with aforementioned classical procedures for identification of related pairs. Figure 4 shows the classification rate as a function of the number variants with MAF 0.50 for four methods: the two IBS-based methods, the (*m, s*) plot and the (*p*_0_, *p*_2_) plot; one IBD-based method, the ($\hat{k}0$, $\hat{k}1$) plot, using the KING estimator (Manichaikul et al., 2010); and the log-ratio PCA approach proposed in this paper. These classification rates were obtained by averaging over 25 replicates of the simulations, for each value of the MAF and the number of variants. It is clear that the log-ratio PCA approach (using three principal components) gives the best classification rates for all relationships. There is little difference in classification rate for third degree relationships, which are relatively more easy to classify. Interestingly, in terms of classification rate the (*m, s*) and (*p*_0_, *p*_2_) plots are seen to be fully equivalent, as they have exactly the same classification rate profile. Posteriorly, we found these statistics to be related by the equations *m* = 1 − *p*_0_ + *p*_2_ and $s=\sqrt{p_{0}\left(1-p_{0}\right)+p_{2}\left(1-p_{2}\right)+2p_{0}p_{2}}$. As expected, classification rate increases with the number of variants. The results suggest that for all four methods 25,000 variants with MAF 0.50 are sufficient to almost perfectly classify PO, FS, second, third, and fourth degree relationships. The difference in classification rate between the log-ratio PCA approach and the conventional methods is larger for the more remote relationships. This simulation concerns a relatively ideal dataset with independent variants and maximally polymorphic variants. For empirical data sets, the independence of the variants can be approximately achieved by LD pruning variants. In practice, many variants have a low MAF. We therefore also investigated the effect of the MAF on the discriminatory power of the different methods, by simulating variants with different MAFs. Figure 5 shows how the classification rate varies as a function of the MAF, using a fixed number of 5.000 bi-allelic polymorphisms. The log-ratio PCA approach, using five principal components, is seen to outperform the classical plots over the full MAF range.

### 3.3. Empirical Data Sets

In this section we use log-ratio PCA for a relatedness study of two genomic data sets. We use the CEU population of the 1,000 genomes project (www.internationalgenome.org, The 1000 Genomes Project Consortium, 2015), whose family relationships have been analyzed in detail by Pemberton et al. (2010), Kyriazopoulou-Panagiotopoulou et al. (2011), Huff et al. (2011), and Stevens et al. (2011; 2012). We also present a relatedness study of the population-based GCAT Genomes for Life project (a cohort study of the genomes of Catalonia, www.genomesforlife.com, Obón-Santacana et al., 2018). For both projects, we used Plink 1.90 (Purcell et al., 2007) for data manipulation, filtering and IBD estimation, and R (R Core Team, 2014) for log-ratio PCA and discriminant analysis.

#### 3.3.1. The CEU Sample

**Figure 6:**
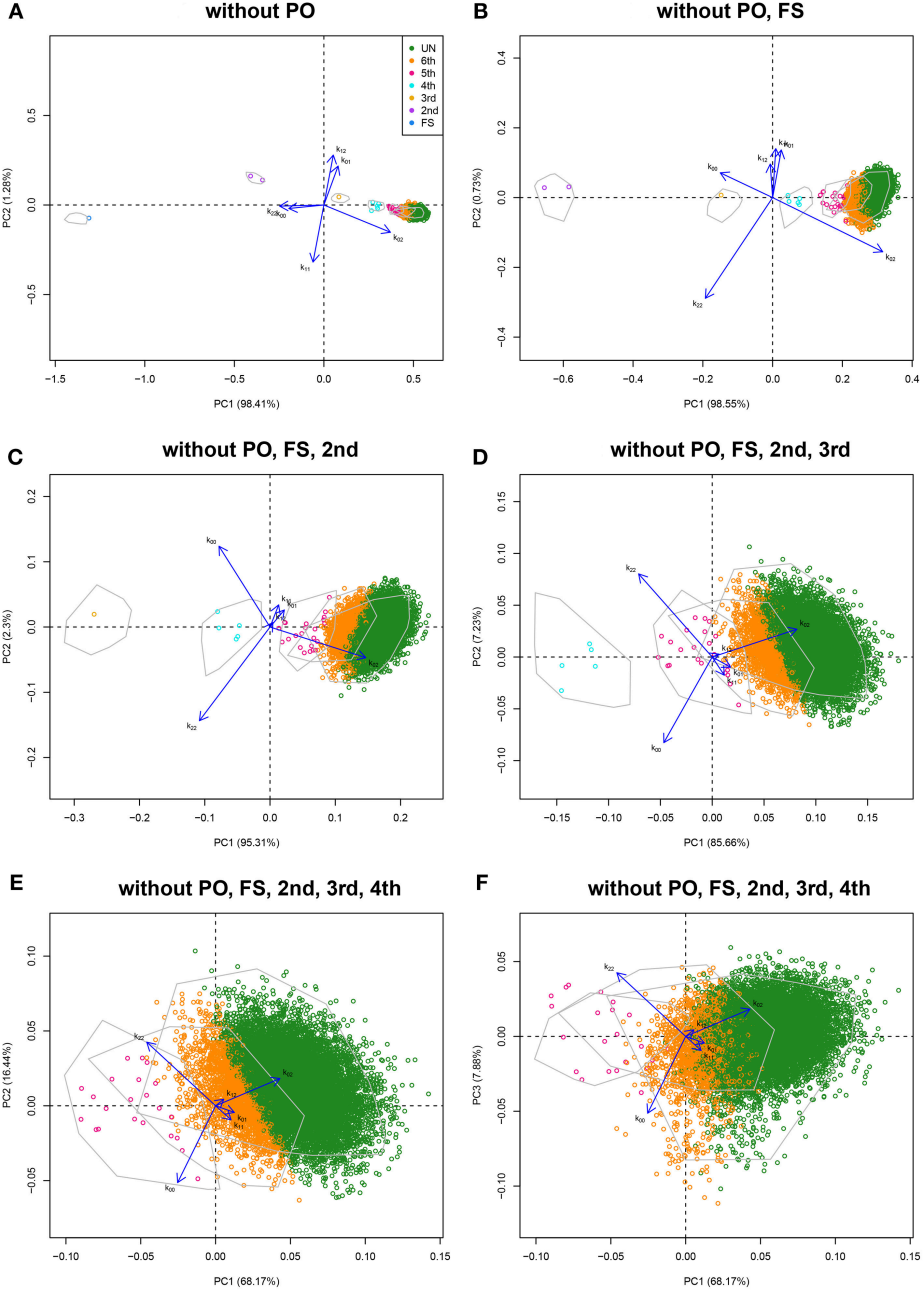
Log-ratio PCA biplots for the CEU sample obtained by peeling and zooming. (A) log-ratio PCA biplot, PO pairs excluded. (B) PO and FS pairs excluded; (C) PO, FS, and AV pairs excluded; (D) PO, FS, AV, and third degree pairs excluded; (E) PO, FS, AV, third and fourth degree pairs excluded (PC1 vs. PC2); (F) PO, FS, AV, third and fourth degree pairs excluded (PC1 vs. PC3). Convex hulls delimit the region of the pairs obtained by simulation.

First and second degree relationships for the CEU population were documented by Pemberton et al. (2010) using IBS methods, and confirmed by Kyriazopoulou-Panagiotopoulou et al. (2011), who used hidden Markov models and suggested additional third and fourth degree relationships. Stevens et al. (2012) used IBD methods confirming the results of Pemberton et al. (2010). We detail the analysis of the CEU panel using log-ratio PCA. Variants were filtered according to missingness (only variants genotyped for all individuals were used), MAF (>0.40) and Hardy-Weinberg equilibrium test result (exact test mid *p*-value >0.05, Graffelman and Moreno, 2013). Variants were LD-pruned with Plink using a sliding window of 50 SNPs with an overlap of 5 SNPs between successive windows, and SNPs are removed from the window until no variants remain that have a squared correlation above 0.20 (Plink option indep-pairwise 50 5 0.2). The final data set contained 31,370 autosomal variants. The CEU panel consists of 165 individuals, mainly PO trios, giving 13,530 possible pairs of individuals. The classical plots of the allele sharing statistics were shown previously in Figure 1, including a log-ratio PCA biplot of all pairs (Figure 1D). We now illustrate the log-ratio PCA approach, using an iterative peel and zoom procedure. Figure 1D showed PO pairs to be outlying in the first dimension, for having a low *k*_02_/*k*_00_ ratio. Theoretically, this ratio is zero for PO pairs, though with large numbers of variants it is non-zero due to mutations and genotyping errors. In fact, the 96 reported PO pairs are easily identified and excluded from the data by filtering with *k*_02_ < 0.005. Log-ratio PCA biplots, obtained by simulation with unrelated individuals of the CEU sample, are shown in Figure 6. The simulated pairs of given relationships are represented by convex hulls, and the projected empirical pairs by open dots that are colored according to their predicted relationship, where the latter are inferred from the posterior probabilities obtained in LDA. The convex hulls delimit the cloud of the positions of the simulated UN, sixth, fifth, fourth, and third degree pairs (using 100 pairs of each). The overall classification rate of the simulated data was 91.4%, using three principal components. Classification rates for third, fourth, fifth, sixth, and UN were, respectively 100, 100, 90, 77, and 90%. Results in Figures 1, 6 suggest the CEU sample has 96 PO pairs, one FS pair, two second degree pairs, one third degree pair, five fourth degree pairs, and many fifth and sixth degree pairs that merge with UN pairs. The analysis without PO pairs in Figure 6A shows the documented FS and AV pairs as outliers in the first dimension, for having high *k*_00_/*k*_02_ and *k*_22_/*k*_02_ ratios. Re-analysis after removal of the FS pair gives Figure 6B, showing the two AV pairs now as strong outliers in the first dimension. Peeling these two pairs, we obtain Figure 6C, with the single documented third degree pair being now the most prominent outlier. Five additional pairs are seen to separate from the UN cloud, and are classified as fourth degree pairs. Re-analysis after peeling off the third degree pair gives a plot with a more clear separation of the fourth degree pairs (Figure 6D). Another set of pairs, presumably of fifth degree, is seen to bud off from the UN cloud more clearly, once the fourth degree pairs are removed from the analysis (Figure 6E), and additional pairs, classified as sixth degree, separate out partly in the third dimension of this analysis. An exploration of the data up to the fifth dimension of the analysis, after peeling the most obvious PO, FS, AV, third, and fourth degree outliers, is shown in Figure S1. These graphs suggest there is information on relatedness up to and including at least the third dimension of the analysis.

The classification of the empirical pairs by *k*_02_ filtering followed by linear discriminant analysis confirmed the 96 PO and the single FS pair relationships described by Pemberton et al. (2010) (results not shown), as well as the additional FC pair reported by Kyriazopoulou-Panagiotopoulou et al. (2011). First and second degree relationships in the CEU sample are easily and almost certainly identified. Much more uncertainty resides in relationships of the third and higher degrees, and for these relationships conflicting inferences are reported in the literature. We therefore carried out a linear discriminant analysis with a simulated training sample containing pairs with a third through sixth degree relationship, as well as UN pairs, and classified all empirical pairs which clearly had no first or second degree relationship. Third and fourth degree relationships uncovered by Kyriazopoulou-Panagiotopoulou et al. (2011) are reported in Table 4, together with the posterior probabilities obtained in our log-ratio PCA approach. We extended Table 4 with additional fifth degree pairs uncovered by log-ratio PCA, for which LDA gave the highest posterior probability. In total, 18 pairs were classified as fifth degree relationship pairs, of which 10 had a posterior probability above 0.95 (marked in bold in Table 4). We tentatively suggest the CEU panel to contain at least ten fifth degree pairs. We found 1,285 sixth degree pairs, but do not report all these pairs in the light of the overlap with the UN cluster and the somewhat poorer classification rate of the sixth degree observed in the simulations.

**Table 4:** Predicted relationships of third (3rd), fourth (4th), and fifth (5th) degree pairs of the CEU sample.

										**Posterior probabilities**								

**Pair**	**ID1**	**Sex**	**ID2**	**Sex**	**Pem**.	**Kyr**.	**Ste**.	**Huf**.	**Predicted**	**3rd**	**4th**	**5th**	**6th**	**UN**	${\hat{k}}_{0}$	${\hat{k}}_{1}$	${\hat{k}}_{2}$	$\hat{\phi }$
1	NA06997	F	NA12801	M	–	FC	FC	–	3rd	1.000	0.000	0.000	0.000	0.000	0.724	0.276	0.000	0.069
2	NA06993	M	NA07022	M	–	4th	–	4th	4th	0.000	1.000	0.000	0.000	0.000	0.870	0.127	0.003	0.033
3	NA06993	M	NA07056	F	–	4th	–	4th	4th	0.000	1.000	0.000	0.000	0.000	0.870	0.130	0.000	0.033
4	NA07031	F	NA12043	M	–	4th	–	–	4th	0.000	1.000	0.000	0.000	0.000	0.845	0.155	0.000	0.039
5	NA12155	M	NA12264	M	–	4th	–	4th	4th	0.000	1.000	0.000	0.000	0.000	0.867	0.133	0.000	0.033
6	NA12760	M	NA12830	F	–	FC	–	–	4th	0.000	1.000	0.000	0.000	0.000	0.855	0.133	0.012	0.039
7	NA06989	F	NA10831	F	–	–	–	–	5th	0.000	0.000	**0.965**	0.035	0.000	0.966	0.026	0.008	0.011
8	NA06989	F	NA12155	M	–	4th	–	–	5th	0.000	0.028	**0.972**	0.000	0.000	0.912	0.088	0.000	0.022
9	NA06991	F	NA07022	M	–	4th	–	–	5th	0.000	0.016	**0.983**	0.000	0.000	0.898	0.102	0.000	0.025
10	NA06994	M	NA12878	F	–	–	–	–	5th	0.000	0.000	0.814	0.185	0.000	0.951	0.041	0.008	0.014
11	NA06994	M	NA12892	F	–	4th	–	5th	5th	0.000	0.000	**0.997**	0.002	0.000	0.925	0.075	0.000	0.019
12	NA07014	F	NA12043	M	–	4th	–	–	5th	0.000	0.000	**0.966**	0.034	0.000	0.950	0.043	0.008	0.015
13	NA07029	M	NA12892	F	–	–	–	–	5th	0.000	0.000	0.563	0.437	0.000	0.942	0.056	0.002	0.015
14	NA07031	F	NA12752	M	–	–	–	–	5th	0.000	0.000	**0.980**	0.020	0.000	0.942	0.053	0.005	0.016
15	NA07031	F	NA12761	F	–	4th	–	–	5th	0.000	0.000	**0.991**	0.009	0.000	0.890	0.110	0.000	0.028
16	NA07055	F	NA10852	F	–	–	–	–	5th	0.000	0.000	0.853	0.147	0.000	0.959	0.040	0.001	0.011
17	NA10830	M	NA12842	M	–	–	–	–	5th	0.000	0.000	0.826	0.174	0.000	0.940	0.060	0.000	0.015
18	NA10852	F	NA10853	M	–	–	–	–	5th	0.000	0.000	0.731	0.269	0.000	0.964	0.033	0.003	0.010
19	NA10852	F	NA11843	M	–	–	–	–	5th	0.000	0.000	0.575	0.425	0.000	0.978	0.019	0.003	0.006
20	NA10863	F	NA12155	M	–	4th	–	–	5th	0.000	0.000	**0.959**	0.041	0.000	0.941	0.054	0.005	0.016
21	NA11843	M	NA11994	M	–	–	–	–	5th	0.000	0.000	0.781	0.219	0.000	0.945	0.055	0.000	0.014
22	NA11992	M	NA12778	F	–	–	–	–	5th	0.000	0.000	0.682	0.318	0.000	0.951	0.050	0.000	0.012
23	NA12752	M	NA12830	F	–	4th	–	–	5th	0.000	0.000	**0.997**	0.003	0.000	0.894	0.106	0.000	0.026
24	NA12760	M	NA12818	F	–	4th	–	–	5th	0.000	0.000	**0.998**	0.002	0.000	0.926	0.074	0.000	0.019
25	NA10831	F	NA12264	M	–	4th	–	–	6th	0.000	0.000	0.094	0.896	0.010	0.963	0.036	0.001	0.010
26	NA11931	F	NA12748	M	–	4th	–	–	6th	0.000	0.000	0.467	0.532	0.001	0.927	0.067	0.006	0.020
27	NA12752	M	NA12818	F	–	4th	–	–	6th	0.000	0.000	0.026	0.946	0.029	0.977	0.022	0.001	0.006

*Third (3rd) and fourth (4th) degree pairs of the CEU sample of the 1000G project as reported by Kyriazopoulou-Panagiotopoulou et al. (2011) and additional detected fifth (5th) degree pairs. Posterior probabilities according to log-ratio PCA combined with LDA. Coding and abbreviations used: sex M = male, F = female; a hyphen (–) indicates the corresponding pair is not annotated or regarded unknown by the corresponding authors; FC, first cousin; Pem., Pemberton et al. (2010); Kyr., Kyriazopoulou-Panagiotopoulou et al. (2011); Ste., Stevens et al. (2012); Huf., Huff et al. (2011)*.

Our results confirm a third degree pair (pair 1 in Table 4) reported by Kyriazopoulou-Panagiotopoulou et al. (2011). We also confirm four of the fourth degree pairs reported by the latter authors (pairs 2–5 in Table 4). However, we also observed considerably incongruence of our results with those of the latter authors. We found an FC pair to be classified as fourth degree (pair 6) by our method and 11 reported fourth degree pairs were classified as fifth or sixth degree. We also compared results with those published by Huff et al. (2011), who estimate recent shared ancestry (ERSA) by using IBD segments. Our work confirms three fourth degree pairs and one fifth degree pair reported by the latter authors, though we found two additional fourth degree pairs, and several fifth degree pairs, which are not confirmed by Huff et al. (2011).

#### 3.3.2. The GCAT Sample

**Figure 7:**
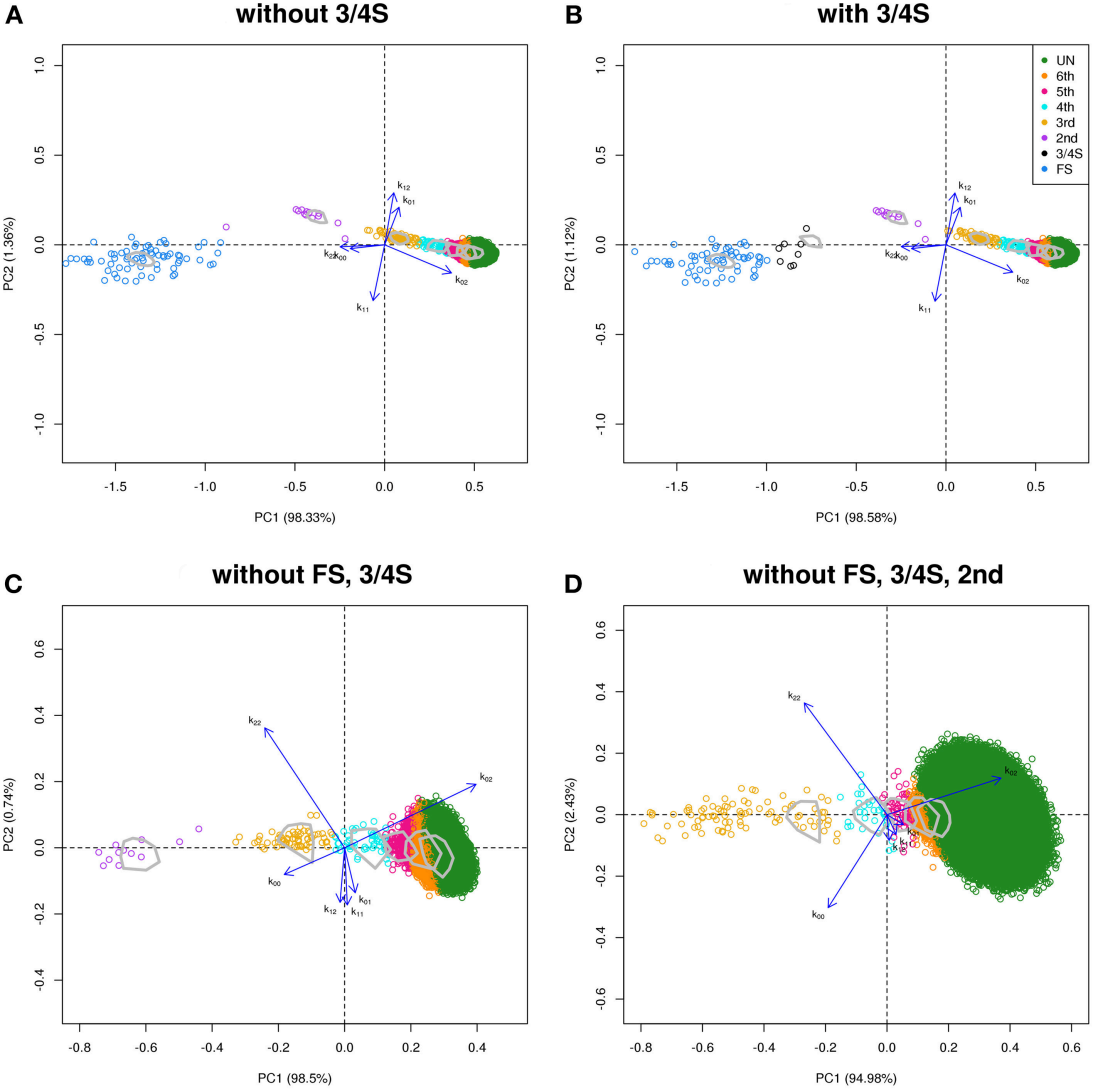
Log-ratio PCA biplot of GCAT sample obtained by peeling and zooming. **(A)** log-ratio PCA biplot, PO and 3/4S pairs excluded. **(B)** 3/4S pairs included; **(C)** FS and 3/4S pairs excluded; **(D)** FS, 3/4S, and second degree pairs excluded. Convex hulls delimit the region of the pairs obtained by simulation.

We use samples from the GCAT Genomes for life project, a cohort study of the genomes of Catalonia (www.genomesforlife.com). GCAT is a prospective cohort study that includes 17,924 participants (40–65 years, release August 2017) recruited from the general population of Catalonia, a Mediterranean region in the northeast of Spain. Participants are mainly part of the Blood and Tissue Bank (BST), a public agency of the Catalan Department of Health. Detailed information regarding the GCAT project is described in Obón-Santacana et al. (2018). We study relatedness of 5,075 GCAT Spanish participants from Caucasian origin using 736,223 SNPs that passed quality control (Galván-Femenía et al., 2018). Inferred relatives of first and second degree were confirmed by the BST public agency, for pairs sharing one surname (PO, second degree pairs) or two surnames (FS pairs), respecting the privacy of the participants. According to the same filtering procedures used in the CEU samples, 26,006 SNPs (MAF > 0.40, LD-pruned, HWE exact mid *p*-value >0.05, and missing call rate 0) were considered for relatedness analysis. PO and MZ pairs potentially having structural zeros were filtered with *k*_02_ < 0.005. Log-ratio PCA biplots representing over twelve million pairs, combined with the classification of the individuals by LDA, and using the peel and zoom procedure, are shown in Figure 7. This analysis shows the different relationships have in general, a larger variability than expected according to the simulated pairs. The FS cluster has a particular high variability, with pairs apparently less related than FS, and pairs stronger related than FS, in comparison with the FS hull. One apparent FS pairs is actually classified as second degree (Figure 7A). This fusion of FS and second degree pairs suggested us that three-quarter siblings might exist in the database and we therefore re-analyzed the data using a training set that included three-quarter siblings. Three-quarter siblings (3/4S) share more IBD alleles than second degree pairs but fewer than FS. 3/4S have one common parent, while their unshared parents can be FS or PO (see Figure S2). Three-quarter siblings have IBD probabilities *k*_0_ = 3/8, *k*_1_ = 1/2, and *k*_2_ = 1/8, such that their kinship coefficient is ϕ = 3/16, below the value ϕ = 1/4 of full siblings. In the re-analysis in Figure 7B, we found 63 FS pairs, 12 2nd pairs, and eight pairs were indeed classified as three-quarter siblings with large posterior probability (see Table 5). Two of these pairs (67, 71) had their kinship coefficient very close to the expected value of ϕ = 3/16. Because Spanish people have both paternal and maternal surnames, three-quarter siblings share both surnames just as siblings do. The pairs classified as 3/4 siblings shared indeed both surnames, confirming these pairs are actually not second degree. Peeling siblings and three-quarter siblings reveals apparent second degree pairs more clearly (Figure 7C). Tentatively peeling second degree pairs brings the third degree pairs in focus (Figure 7D), and in this analysis we find 174 third, 66 fourth, 31 fifth, and 3,517 sixth degree pairs. Further peeling is difficult as the different clusters increasingly merge. In log-ratio PCA the clusters representing the different relationships have more elliptical shapes that separate better. Note that the number of pairs classified as sixth degree decreases as the lower degree relationships are peeled in the analysis.

**Table 5:** Predicted FS and 3/4S relationships of the GCAT sample.

						**Posterior probabilities**										

**Pair**	**ID1**	**Sex**	**ID2**	**Sex**	**Predicted**	**FS**	**3/4S**	**2nd**	**3rd**	**4th**	**5th**	**6th**	**UN**	$\hat{k}0$	$\hat{k}1$	$\hat{k}2$	$\hat{\phi }$
1	REL_00339	F	REL_02473	F	FS	1	0	0	0	0	0	0	0	0.254	0.479	0.266	0.253
2	REL_04741	F	REL_02513	F	FS	1	0	0	0	0	0	0	0	0.187	0.518	0.295	0.277
3	REL_00601	M	REL_02989	F	FS	1	0	0	0	0	0	0	0	0.190	0.508	0.303	0.278
4	REL_02339	M	REL_02391	M	FS	1	0	0	0	0	0	0	0	0.267	0.442	0.290	0.256
5	REL_03977	M	REL_01080	M	FS	1	0	0	0	0	0	0	0	0.222	0.538	0.240	0.255
6	REL_03220	F	REL_04615	F	FS	1	0	0	0	0	0	0	0	0.311	0.460	0.229	0.230
7	REL_04475	F	REL_04218	M	FS	1	0	0	0	0	0	0	0	0.248	0.514	0.237	0.247
8	REL_01150	F	REL_04384	F	FS	1	0	0	0	0	0	0	0	0.258	0.490	0.253	0.249
9	REL_01285	M	REL_03761	F	FS	1	0	0	0	0	0	0	0	0.237	0.496	0.267	0.257
10	REL_04693	F	REL_00797	F	FS	1	0	0	0	0	0	0	0	0.310	0.471	0.220	0.228
11	REL_00383	F	REL_03293	M	FS	1	0	0	0	0	0	0	0	0.254	0.530	0.216	0.241
12	REL_03212	M	REL_02516	F	FS	1	0	0	0	0	0	0	0	0.275	0.526	0.199	0.231
13	REL_00282	F	REL_04918	F	FS	1	0	0	0	0	0	0	0	0.247	0.440	0.313	0.267
14	REL_04616	F	REL_02777	F	FS	1	0	0	0	0	0	0	0	0.279	0.471	0.250	0.243
15	REL_00792	F	REL_00954	M	FS	1	0	0	0	0	0	0	0	0.262	0.509	0.229	0.242
16	REL_03627	F	REL_03315	F	FS	1	0	0	0	0	0	0	0	0.148	0.549	0.302	0.288
17	REL_00872	F	REL_01784	F	FS	1	0	0	0	0	0	0	0	0.252	0.528	0.221	0.242
18	REL_03442	F	REL_04510	F	FS	1	0	0	0	0	0	0	0	0.216	0.512	0.272	0.264
19	REL_01924	F	REL_00727	M	FS	1	0	0	0	0	0	0	0	0.236	0.449	0.315	0.270
20	REL_04704	F	REL_00804	M	FS	1	0	0	0	0	0	0	0	0.168	0.523	0.308	0.285
21	REL_04494	M	REL_00931	M	FS	1	0	0	0	0	0	0	0	0.280	0.492	0.228	0.237
22	REL_04439	F	REL_01640	F	FS	1	0	0	0	0	0	0	0	0.264	0.430	0.306	0.260
23	REL_00504	M	REL_04718	F	FS	1	0	0	0	0	0	0	0	0.243	0.505	0.252	0.252
24	REL_01624	F	REL_00750	F	FS	1	0	0	0	0	0	0	0	0.191	0.508	0.301	0.278
25	REL_01524	F	REL_03272	F	FS	1	0	0	0	0	0	0	0	0.232	0.511	0.257	0.256
26	REL_00769	M	REL_04746	F	FS	1	0	0	0	0	0	0	0	0.225	0.566	0.208	0.246
27	REL_01654	M	REL_03485	M	FS	1	0	0	0	0	0	0	0	0.282	0.432	0.285	0.251
28	REL_01564	F	REL_03827	F	FS	1	0	0	0	0	0	0	0	0.316	0.427	0.258	0.236
29	REL_03944	M	REL_03475	F	FS	1	0	0	0	0	0	0	0	0.231	0.542	0.227	0.249
30	REL_01888	M	REL_04360	M	FS	1	0	0	0	0	0	0	0	0.247	0.543	0.210	0.241
31	REL_00824	F	REL_00213	F	FS	1	0	0	0	0	0	0	0	0.221	0.446	0.332	0.278
32	REL_03838	F	REL_02496	F	FS	1	0	0	0	0	0	0	0	0.310	0.446	0.245	0.234
33	REL_00122	M	REL_01902	F	FS	1	0	0	0	0	0	0	0	0.286	0.494	0.220	0.233
34	REL_04592	F	REL_04600	F	FS	1	0	0	0	0	0	0	0	0.305	0.485	0.211	0.227
35	REL_00284	M	REL_02444	F	FS	1	0	0	0	0	0	0	0	0.278	0.511	0.211	0.233
36	REL_03395	F	REL_02694	F	FS	1	0	0	0	0	0	0	0	0.224	0.522	0.254	0.257
37	REL_02718	M	REL_02913	M	FS	1	0	0	0	0	0	0	0	0.218	0.479	0.303	0.271
38	REL_00968	M	REL_01577	F	FS	1	0	0	0	0	0	0	0	0.257	0.451	0.292	0.259
39	REL_01502	M	REL_03665	M	FS	1	0	0	0	0	0	0	0	0.312	0.477	0.211	0.225
40	REL_03904	F	REL_04994	F	FS	1	0	0	0	0	0	0	0	0.250	0.502	0.248	0.249
41	REL_02208	F	REL_03486	F	FS	1	0	0	0	0	0	0	0	0.231	0.460	0.310	0.270
42	REL_02208	F	REL_01630	F	FS	1	0	0	0	0	0	0	0	0.177	0.516	0.307	0.283
43	REL_03486	F	REL_01630	F	FS	1	0	0	0	0	0	0	0	0.170	0.502	0.327	0.289
44	REL_00340	F	REL_04294	F	FS	1	0	0	0	0	0	0	0	0.210	0.525	0.265	0.264
45	REL_02899	M	REL_01707	F	FS	1	0	0	0	0	0	0	0	0.285	0.454	0.261	0.244
46	REL_03001	F	REL_04111	F	FS	1	0	0	0	0	0	0	0	0.230	0.481	0.289	0.265
47	REL_00634	M	REL_03507	M	FS	1	0	0	0	0	0	0	0	0.203	0.508	0.289	0.272
48	REL_02905	F	REL_02575	F	FS	1	0	0	0	0	0	0	0	0.252	0.517	0.231	0.245
49	REL_01016	M	REL_00887	M	FS	1	0	0	0	0	0	0	0	0.243	0.496	0.260	0.254
50	REL_03151	M	REL_02204	F	FS	1	0	0	0	0	0	0	0	0.235	0.503	0.263	0.257
51	REL_04466	F	REL_02680	F	FS	1	0	0	0	0	0	0	0	0.313	0.427	0.260	0.237
52	REL_03607	M	REL_00319	F	FS	1	0	0	0	0	0	0	0	0.299	0.491	0.210	0.228
53	REL_01083	F	REL_01704	F	FS	1	0	0	0	0	0	0	0	0.182	0.567	0.251	0.267
54	REL_04427	F	REL_02635	F	FS	1	0	0	0	0	0	0	0	0.264	0.545	0.191	0.232
55	REL_01546	M	REL_03566	F	FS	1	0	0	0	0	0	0	0	0.212	0.525	0.263	0.263
56	REL_01450	M	REL_01960	M	FS	1	0	0	0	0	0	0	0	0.259	0.514	0.227	0.242
57	REL_03310	M	REL_03659	F	FS	1	0	0	0	0	0	0	0	0.259	0.559	0.182	0.231
58	REL_03880	M	REL_04789	F	FS	1	0	0	0	0	0	0	0	0.271	0.503	0.226	0.239
59	REL_01264	M	REL_04751	F	FS	1	0	0	0	0	0	0	0	0.183	0.518	0.299	0.279
60	REL_04529	F	REL_04492	F	FS	1	0	0	0	0	0	0	0	0.279	0.498	0.223	0.236
61	REL_03388	F	REL_02608	F	FS	1	0	0	0	0	0	0	0	0.216	0.497	0.287	0.268
62	REL_00009	F	REL_02335	F	FS	1	0	0	0	0	0	0	0	0.233	0.548	0.218	0.246
63	REL_04405	M	REL_03949	M	FS	1	0	0	0	0	0	0	0	0.262	0.523	0.215	0.238
64	REL_02752	F	REL_04859	F	3/4S	0	1	0	0	0	0	0	0	0.342	0.457	0.201	0.215
65	REL_01344	M	REL_02408	F	3/4S	0	1	0	0	0	0	0	0	0.361	0.439	0.200	0.210
66	REL_00083	M	REL_02333	M	3/4S	0	1	0	0	0	0	0	0	0.326	0.520	0.154	0.207
67	REL_03803	F	REL_02343	M	3/4S	0	1	0	0	0	0	0	0	0.349	0.510	0.140	0.198
68	REL_03924	M	REL_03023	F	3/4S	0	1	0	0	0	0	0	0	0.366	0.464	0.170	0.201
69	REL_04189	M	REL_00775	M	3/4S	0	1	0	0	0	0	0	0	0.367	0.427	0.206	0.210
70	REL_03150	F	REL_01804	F	3/4S	0	1	0	0	0	0	0	0	0.323	0.505	0.172	0.212
71	REL_03969	M	REL_00271	M	3/4S	0	1	0	0	0	0	0	0	0.342	0.560	0.098	0.189

*FS and 3/4S pairs of the GCAT sample. Predicted relationships and posterior probabilities according to a log-ratio PCA combined with LDA. Coding and abbreviations used: sex M, male; F, female; $\hat{\phi }$, estimated kinship coefficient*.

For all simulated and empirical data sets studied above, the first principal component in the log-ratio PCA's is seen to strongly correlate with the kinship coefficient. The corresponding scatterplots and correlation coefficients are shown in Figure S3. The first principal component is clearly interpretable as a relatedness index. In Figures 6A, 7A (without PO), the biplot vectors show that the first component separates homogeneous homozygote pairs (AA & AA; BB & BB) from heterogeneous homozygote pairs (AA & BB). The second principal component separates double heterzygote pairs from single heterozygote pairs. When FS pairs are removed, the second principal component changes, and reflects a contrast between pairs with heterozygotes and without heterozygotes.

## 4. Discussion

We have developed a log-ratio PCA based procedure that can be used for uncovering cryptic relatedness in homogeneous populations. Simulations show the procedure has a better classification rate than the classical IBS and IBD based approaches. The log-ratio PCA approach exploits the compositional nature of genotype sharing counts over variants, and can potentially use five dimensions for analysis, whereas the classical approaches collapse the data in two dimensions. The analysis of the CEU sample has led to the identification of a set of hitherto unreported pairs for which a fifth degree relationship is highly plausible (Table 4). Our conclusion is that log-ratio PCA, combined with LDA, increases the resolution of relationship discrimination. The classification rate for 6th degree pairs can still be improved if more than 35,000 independent MAF 0.50 variants would be used (see Figure 4). The (*p*_0_, *p*_2_), (*m, s*), and ($\hat{k}0$, $\hat{k}1$) scatterplots display estimates in a constrained space (Galván-Femenía et al., 2017), where Euclidean distances between points cannot be safely interpreted. This is particularly true for the higher degree relationships that merge toward the vertex of the triangular region inside the scatterplot. Log-ratio PCA, besides using more dimensions, frees the data of the unit sum constraint, and clearly enhances the discrimination of the higher degree relationships. We have compared our log-ratio based procedure with some basic procedures used in relatedness research. Its performance could be further explored in a more extensive comparison that includes IBD-segment based methods, such as the comprehensive study reported by Ramstetter et al. (2017).

The analysis of the GCAT samples shows, for almost all relationship categories, larger variability in the relationship clusters than would be expected under strict Mendelian sampling of alleles from unrelated individuals. This excess variability can, at least in part, be explained by the presence of additional relatedness between (unobserved) close relatives of the individuals in the database. This leads to increased autozygosity, which is a characteristic of more endogamous populations. The occurrence of three-quarter siblings is just a particular instance of this phenomenon. Consequently, the degree of relatedness of two individuals tends to become a continuous variable, which is increasingly hard to discretize into the standard relationship categories.

The simulated reference data sets were obtained by resampling genetic variants independently, and this does not take linkage disequilibrium (LD) and recombination into account (Hill and Weir, 2011). If the genotype data is phased, a biologically more realistic simulated data set can be obtained by sampling haplotypes. We have avoided this issue by LD pruning the data base prior to resampling, so removing tightly correlated markers. The reference data set is therefore constructed on the basis of a subset of variants that can expected to be approximately independent. This subset is then used as the basis for relationship estimation. This procedure has the advantage that it avoids potential additional uncertainty generated by using a phasing algorithm. However, the proposed procedure may be improved in the future by accounting for haplotype structure and recombination. The pruning threshold used in our method (0.20) is a compromise between precision and satisfying the independence assumption. A larger value will admit more variants and can increase the resolution, but due to correlation between variants it will invalidate the independence assumption used to generate the reference set of related pairs.

The proposed method for classifying pairs combining log-ratio PCA and discriminant analysis is seen to perform well with both simulated and empirical data. The sampling of artificially related pairs from the observed data requires a considerable number of approximately unrelated individuals to be present in the database. We therefore suggest the method to be used for large samples with thousands of individuals, where such a substantial subset of unrelated individuals can be identified. This is probably not an obstacle for the use of our method, as increasingly large samples are being used in epidemiological genomics. The sampling of artificial pairs from the observed data respects the allele frequency distribution of the original data, and provide reference areas for the different relationships given the allele frequencies of the observed data. Note that with only one hundred simulated pairs of each relationship, we build a classifier that can be used to classify millions of pairs. Our method is computationally feasible for over 5,000 individuals and 26,000 variants like in the GCAT sample. Most of the computation time is spent on the projection of the empirical pairs onto the reference structure, and these computations could easily be parallelized. Many public repositories of genomic data are currently available, but without recruitment and relatedness information, and for which the relatedness techniques discussed in this paper could be usefully applied.

The log-ratio transformation in Equation (1) does not admit zeros for the genotype sharing counts. In theory MZ pairs have *k*_10_ = *k*_20_ = *k*_21_ = 0, and PO pairs have *k*_20_ = 0. In practice, due to the summing over large numbers of variants, zeros are almost never observed as a consequence of some genotyping error and incidental mutations. If a few zero counts are observed, a replacement by 1 or 0.5 can eventually be used in order to proceed with the analysis. If there is a substantial amount of zeros, a ratio-preserving multiplicative replacement (Fry et al., 2000; Martín-Fernández et al., 2003) or a Bayesian procedure (Martin-Fernandez et al., 2015) are recommended. The zero problem is well-known in compositional data analysis, and a distinction is usually drawn between structural and rounding zeros (Martín-Fernández et al., 2003, 2011). In principle, MZ and PO pairs have structural zeros. However, MZ and PO pairs are the most easily detected relationships, and are easily dealt with separately, prior to applying the log-ratio transformation to the data. Exclusion of the relationships up to the second or third degree is in fact desirable if possible, as it will allow the study of the more remote relationships at higher resolution.

We recommend the use of discriminant analysis in allele-sharing studies as employed in this paper. The posterior probabilities of the different relationships give a quantitative criterion for deciding upon which relationship is most likely for a given pair of individuals. In allele sharing studies this decision is mostly made graphically by inspecting a (*p*_0_, *p*_2_) plot in IBS studies, or a ($\hat{k}0$, $\hat{k}1$) plot in IBD studies. We note that these posterior probabilities differ from those used in a standard discriminant analysis, in the sense that they are affected by additional uncertainty generated by using a training set obtained by a resampling of the observed data.

Applications of IBD based methods typically employ three Cotterman coefficients that are constrained to sum one, and therefore represent relatedness in only two dimensions. However, IBD based methods can estimate additional Jacquard coefficients (Milligan, 2003) and thus potentially exploit more dimensions than is usually done in practice.

The current paper is focused on homogeneous populations. If population substructure exists, then log-ratio PCA can be expected to separate the different populations in its biplot. Methods that address substructure (distant relatedness) and family relationships (recent relatedness) jointly have been developed (Manichaikul et al., 2010; Conomos et al., 2015). Population substructure can be accounted for by using only variants with low weights on the first components for a relatedness analysis, as is done in the UK Biobank project (Bycroft et al., 2018), as the first components mostly capture substructure. In future work, the usefulness of the log-ratio PCA approach for the joint study of remote and recent relatedness could be further explored.

## Software Availability

R code (R Core Team, 2014) implementing the logratio kinship biplot proposed in this paper is available online at github.com/ivangalvan/LR-kinbiplot.

## Ethics Statement

Our study does use data from human subjects, but concerns data that is available in public repositories.

## Author Contributions

JG and IG contributed equally to this paper, where JG conceived the methodology and wrote the paper. IG developed computer programs, ran simulations, and performed data analysis. RdC supervised GCAT data analysis. RdC and CBV proof-read the manuscript. All authors contributed to the improvement of the paper.

### Conflict of Interest Statement

The authors declare that the research was conducted in the absence of any commercial or financial relationships that could be construed as a potential conflict of interest.
