# Supplementary material for: A Log-Ratio Biplot Approach for Exploring Genetic Relatedness Based on Identity by State
Source: Front Genet. 2019 Apr 24;10:341. doi: 10.3389/fgene.2019.00341 (PMC6491861; doi:10.3389/fgene.2019.00341)
Supplement: Supplementary file 1 [file Data_Sheet_1.pdf]

Supplementary Material

	True relationship							
Estimated	FS	3/4S	2ND	3RD	4TH	5TH	6TH	UN
FS	100	0	0	0	0	0	0	0
3/4S	0	100	0	0	0	0	0	0
2ND	0	0	100	0	0	0	0	0
3RD	0	0	0	100	0	0	0	0
4TH	0	0	0	0	98.12	1.76	0	0
5TH	0	0	0	0	1.88	84.32	13.92	0.08
6TH	0	0	0	0	0	13.68	72.16	13.72
UN	0	0	0	0	0	0.24	13.92	86.2

Table S1. Confusion matrix of the simulations. Percentages shown are averaged over 25 simulations with 35.000 SNPs with MAF 0.50.

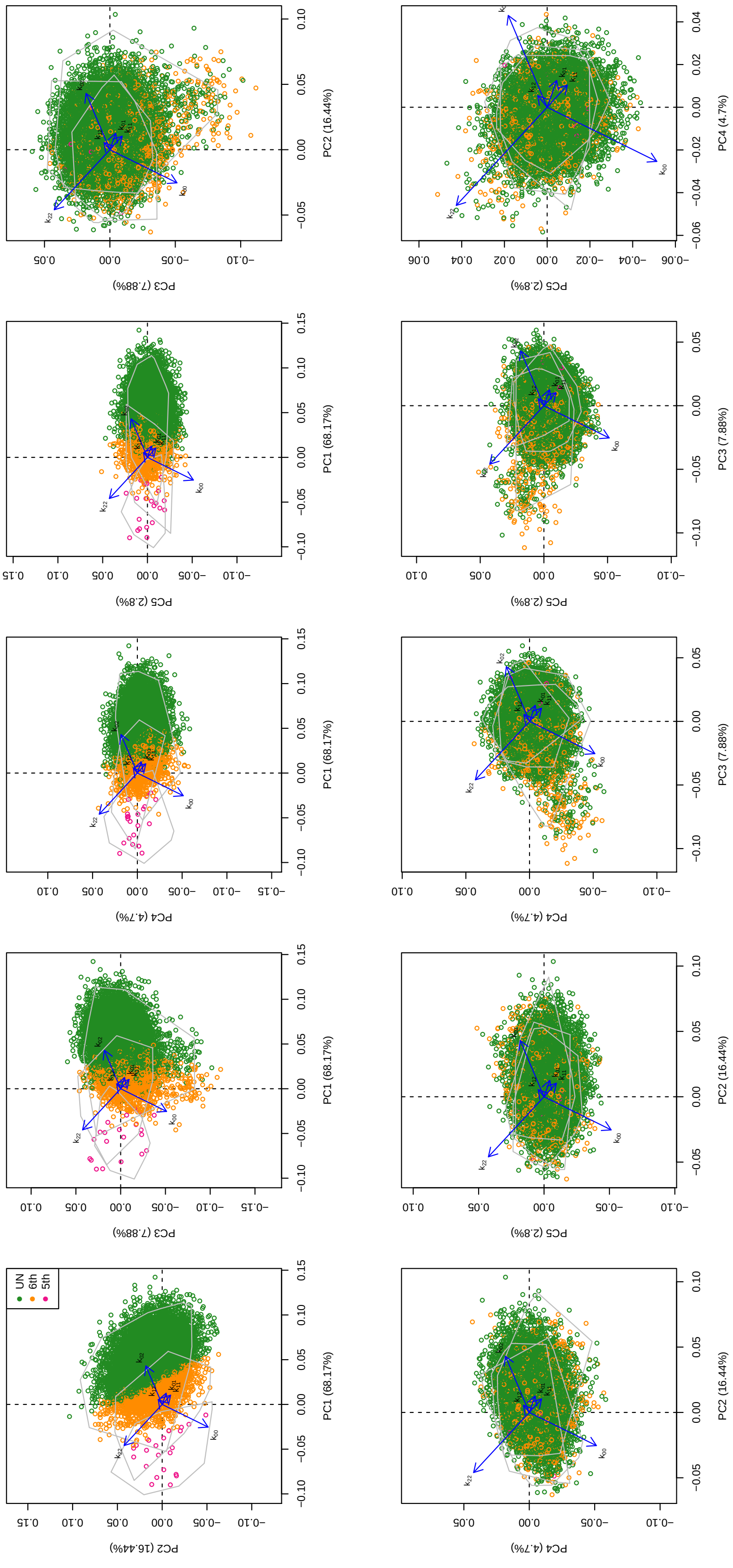

Figure S1. Exploration of fifth, sixth and UN pairs for all five log-ratio principal components.

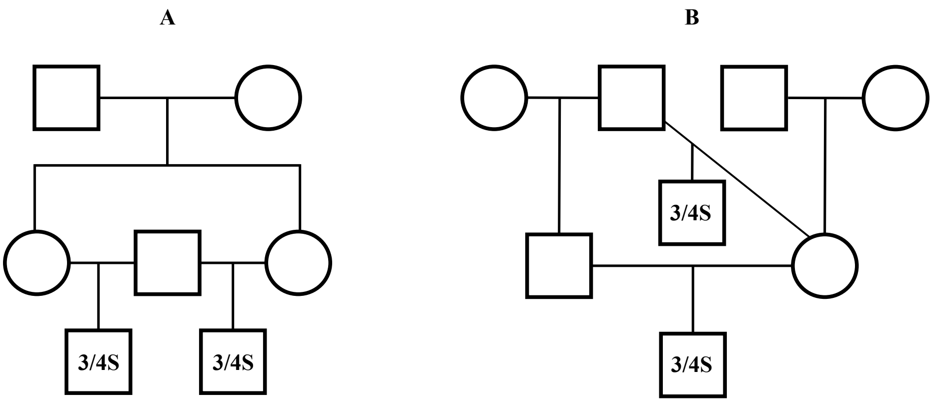

Figure S2. Two pedigree structures (A & B) for three-quarter siblings.

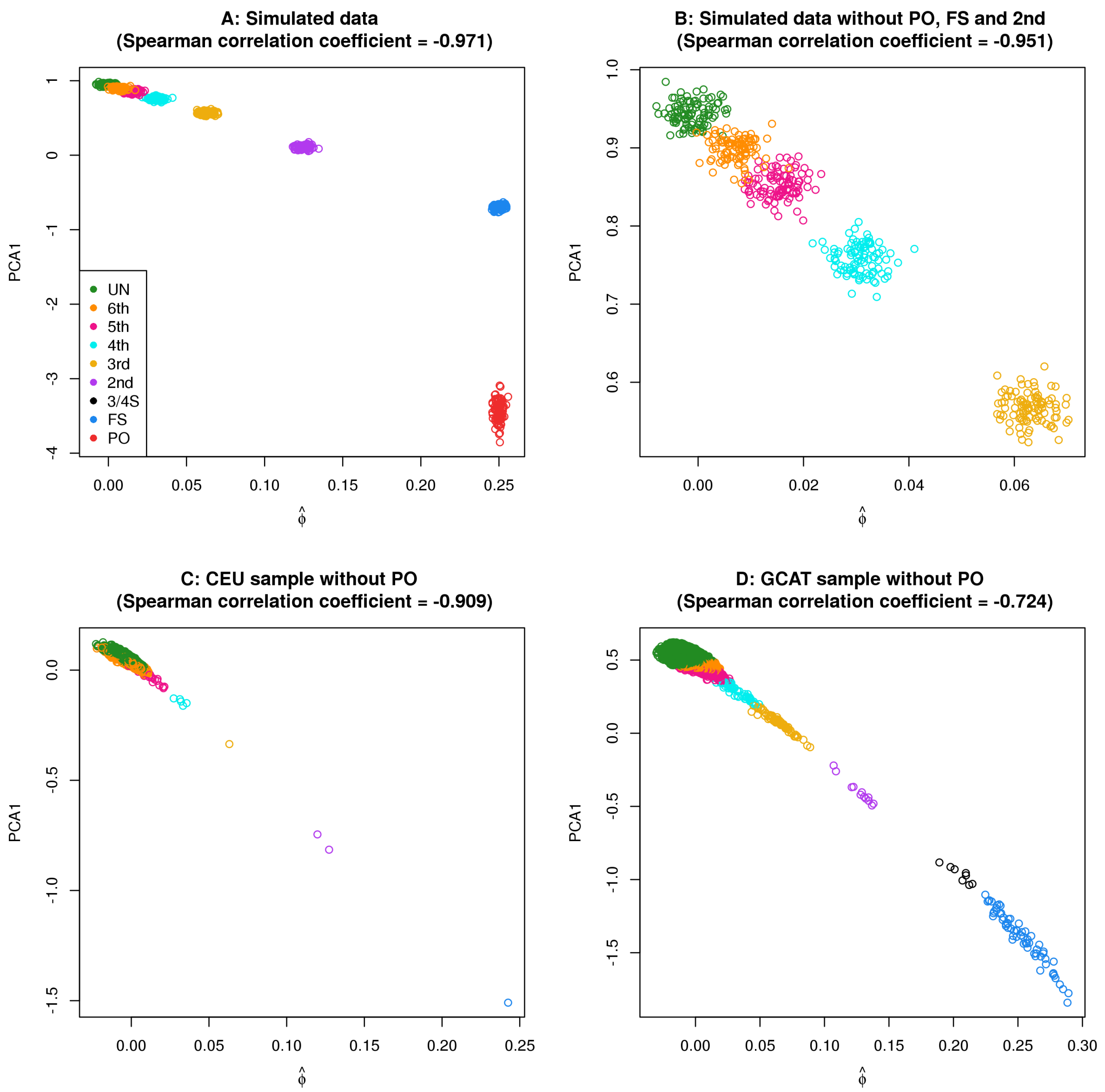

Figure S3. Relation between the first log-ratio principal component and the kinship coefficient for simulated and empirical datasets.
